# Charting the course of ferroptosis research in lung cancer: Insights from a bibliometric analysis

**DOI:** 10.1016/j.heliyon.2024.e24810

**Published:** 2024-01-19

**Authors:** Huatao Zhou, Yu Mao, Zijing Zhou

**Affiliations:** a Department of Cardiovascular Surgery, The Second Xiangya Hospital, Central South University, Middle Renmin Road 139, Changsha, 410011, China; b Department of Thyroid Surgey, The Second Xiangya Hospital, Central South University, Middle Renmin Road 139, Changsha, 410011, China; c Department of Pulmonary and Critical Care Medicine, The Second Xiangya Hospital, Central South University, Middle Renmin Road 139, 410011, Changsha, China

**Keywords:** Ferroptosis, Lung cancer, Bibliometric analysis, CiteSpace, VOSviewer

## Abstract

**Background:**

Lung cancer, a major cause of cancer-related mortality globally, necessitates innovative therapeutic strategies. Ferroptosis, an iron-dependent, non-apoptotic cell death form, has risen as a crucial therapeutic target. This study aims to conduct a comprehensive bibliometric analysis of ferroptosis in lung cancer, highlighting principal research trends, influential publications, and prospective future directions.

**Methods:**

This study utilized bibliometric tools such as VOSviewer, CiteSpace, and the R package “bibliometrix” to thoroughly analyze 488 articles on ferroptosis in lung cancer from 2014 to October 2023. Data from the Web of Science Core Collection were analyzed to determine spatial and temporal trends, identify prominent authors and seminal works, and uncover emerging hotspots and frontiers of the field. The literature was segmented into coherent thematic groups through cluster analysis.

**Results:**

Our analysis revealed a significant exponential growth in publications from 2019 to 2023, mirroring the increasing interest in this area. Predominantly, the influential research was published in high-impact journals, with Scott J. Dixon's works being the most cited. The study identified four primary research themes: Lung Cancer Specifics; Biomarker Identification and Prognosis; Cellular Death Mechanisms and Metabolic Regulation; and Cancer Stem Cells and Therapeutic Resistance. Recent studies have increasingly focused on areas such as the immune microenvironment and mitochondrial dysfunction. Furthermore, the analysis highlighted the field's global collaborative nature, with significant contributions from China, the USA, and Germany.

**Conclusion:**

This extensive bibliometric analysis emphasizes the growing importance of ferroptosis in lung cancer research. The identified themes and emerging topics underline the field's complexity and suggest new research avenues. This study promotes a holistic research approach, advocating for the exploration of innovative ferroptosis-targeting therapies that could revolutionize lung cancer treatment.

## 1 Introduction

Ferroptosis, a novel form of programmed cell death characterized by iron-dependent lipid peroxide accumulation [1,2], was first identified by Professor Brent R. Stockwell of Columbia University in 2012 [3]. Morphologically, ferroptosis is distinguished by reduced mitochondrial size, increased mitochondrial membrane density, diminished or absent mitochondrial cristae, uncondensed chromatin, and a preserved cellular membrane, leading to a complete spherical cell morphology [4,5]. Ferroptosis susceptibility is intricately linked to various biological processes, including amino acid metabolism, iron, polyunsaturated fatty acids, and the biosynthesis of glutathione, phospholipids, NADPH, and CoQ10 [[6], [7], [8]]. Notably implicated in a range of pathologies such as neurodegenerative diseases, cancer, ischemia-reperfusion injuries, and renal degeneration [[9], [10], [11], [12], [13]], ferroptosis holds significant promise for the development of diverse pharmacological therapies.

The complexity and heterogeneity of lung cancer necessitate a comprehensive approach to understanding its pathophysiology. Ferroptosis offers a unique perspective in examining lung cancer's vulnerability and resilience to different treatments. Despite being a recent discovery, ferroptosis has been linked to key aspects of lung cancer biology, including oxidative stress responses, iron metabolism, and interactions with other cell death forms [14,15]. These insights are crucial for developing novel therapeutic strategies and improving clinical outcomes.

For this bibliometric analysis, we employed advanced tools, namely CiteSpace, VOSviewer, and Bibliometrix, to systematically review and analyze the literature. This approach allowed us to quantitatively and qualitatively evaluate contributions from researchers, institutions, and countries, as well as visualize collaboration networks and identify key trends and hotspots in ferroptosis research relevant to lung cancer. Our study aims to provide a comprehensive overview of ferroptosis in lung cancer, filling a critical research gap. By integrating insights from various fields, we seek to unravel the complex dynamics of ferroptosis in lung cancer and establish a foundation for future research that could transform clinical practice and patient care.

## 2 Methods

### 2.1 Search strategy

**Fig. 1:**
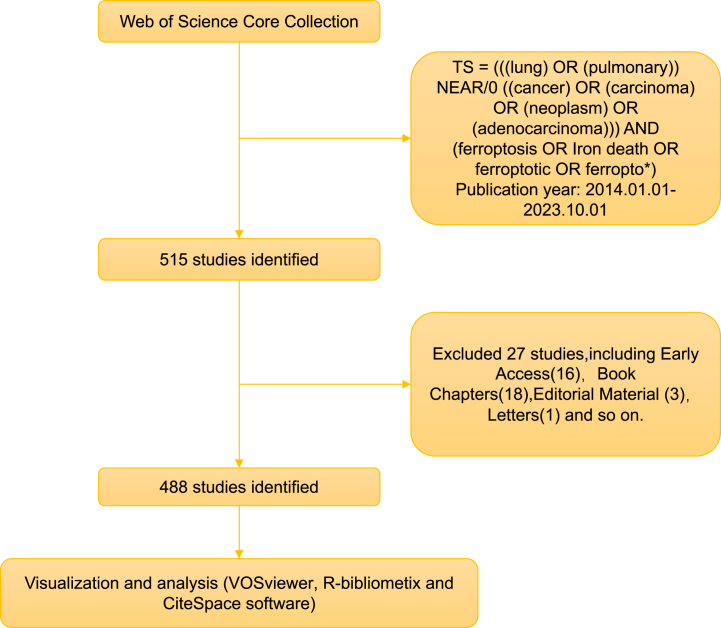
Process for literature screening.

Our search included literature from January 1, 2014, to October 1, 2023, using the following query: TI = (ferroptosis OR Iron death OR ferroptotic OR ferropto*) AND TI= (((lung) OR (pulmonary)) NEAR/0 ((cancer) OR (carcinoma) OR (neoplasm) OR (adenocarcinoma))). Two independent reviewers performed the search, resolving discrepancies through discussion or third-party consultation. The complete dataset was acquired in TEXT format on October 15, 2023, and our data collection and analysis methodology is outlined in Fig. 1.

### 2.2 Data analysis

We utilized VOSviewer (version 1.6.19) for its effectiveness in extracting insights from a vast array of publications [16], commonly used to construct networks of collaboration, co-citation, and keyword co-occurrence [17,18]. This study employed VOSviewer to analyze countries, institutions, journals, co-cited journals, authors, and keyword co-occurrence patterns.

Moreover, CiteSpace (version 6.2.R4), developed by Professor Chen C, was utilized for its capabilities in bibliometric analysis and visualization [[19], [20], [21]]. CiteSpace was instrumental in generating dual-map overlays of journals and conducting comprehensive analyses of references and keyword clusters.

To map the global distribution of publications on ferroptosis in lung cancer, we used the R package “bibliometrix” (version 3.2.1), as detailed at https://www.bibliometrix.org [22]. Data on journal impact factors and quartiles were obtained from the Journal Citation Reports (JCR) 2022, and Microsoft Excel 2021 was crucial for quantitative analysis of the publications.

## 3 Results

### 3.1 Publication trends of ferroptosis in lung cancer research

**Fig. 2:**
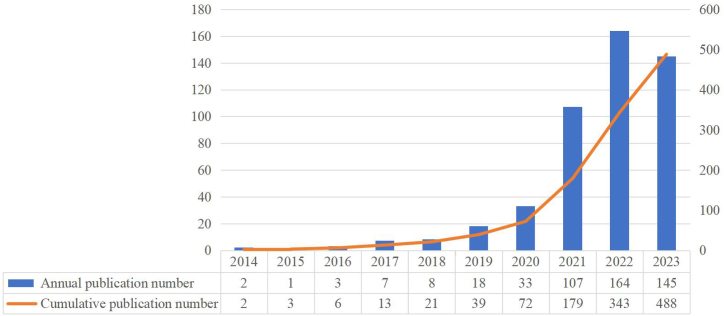
Annual research output on ferroptosis in lung cancer.

Our bibliometric study identified 488 studies on ferroptosis in lung cancer from 2014 to October 1, 2023, comprising 390 articles and 98 reviews. The annual publication growth analysis highlighted two phases: Phase I (2014–2018), with a modest publication count indicative of the nascent stage of ferroptosis research, and Phase II (2019–2023), marked by a substantial increase in publications, signaling major advancements and heightened academic interest. The year-over-year growth rate between 2018 and 2019 was 125 %, signaling a significant escalation in research output (Fig. 2).

### 3.2 Geographical and institutional distribution

**Fig. 3:**
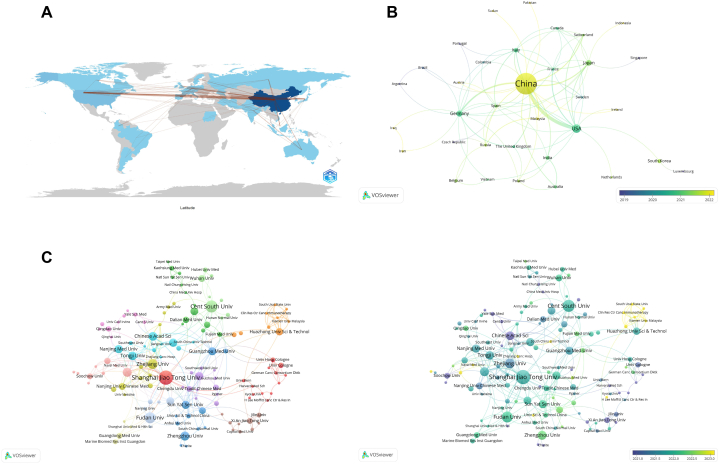
Geographical collaboration map (A), visualization of collaborating countries (B), research institutions (C), and analysis of their publication counts in recent years (D) related to ferroptosis in lung cancer.

This study encompasses contributions from 37 countries and 654 institutions. Notably, China leads in literature output, accounting for approximately two-thirds (67.99 %) of total publications, followed by the United States (8.13 %) and Germany (3.63 %) (Fig. 3A, Table 1). Additionally, publications from China are predominantly more recent, as indicated in Fig. 3B. These statistics underscore China's prominent role in ferroptosis research, highlighted by the highest centrality index of 0.49 among all countries.

**Table 1:** Top 10 countries and institutions with the most documents on research of ferroptosis in lung cancer.

Rank	Country	Centrality	Count(%)	Institution	Centrality	Count(%)
1	China	0.49	393(67.99 %)	Shanghai Jiao Tong University	0.12	29(2.46 %)
2	USA	0.47	47(8.13 %)	Central South University	0.08	24(2.04 %)
3	Germany	0.47	21(3.63 %)	Chinese Academy of Sciences	0.16	19(1.61 %)
4	Japan	0.24	19(3.29 %)	Fudan University	0.02	19(1.61 %)
5	Italy	0.06	11(1.90 %)	Zhejiang University	0.06	16(1.36 %)
6	South Korea	0.10	9(1.56 %)	Zhengzhou University	0.04	14(1.19 %)
7	India	0.02	8(1.38 %)	Shanghai University of Traditional Chinese Medicine	0.02	14(1.19 %)
8	Spain	0.15	5(0.87 %)	Tongji University	0.03	13(1.10 %)
9	Belgium	0.01	5(0.87 %)	Guangzhou Medical University	0.22	12(1.02 %)
10	Australia	0.00	5(0.87 %)	Sichuan University	0.07	12(1.02 %)

At the institutional level, Shanghai Jiao Tong University ranks first with 29 publications (2.46 %). Central South University, the Chinese Academy of Sciences, and Fudan University also feature prominently. Despite the quantity of Chinese publications, their centrality indices are generally lower, suggesting a diverse research landscape within China (Fig. 3C–Table 1).

Other Asian countries, like South Korea and India, along with European nations such as Italy, Spain, and Belgium, are also demonstrating significant research activity. Australia, on par with Belgium and Spain in publication numbers (each with 5 papers, constituting 0.87 % of the total), has a centrality index of 0, indicating a less pronounced impact in this specific research area (Table 1).

### 3.3 Journals and Co-cited journals

**Fig. 4:**
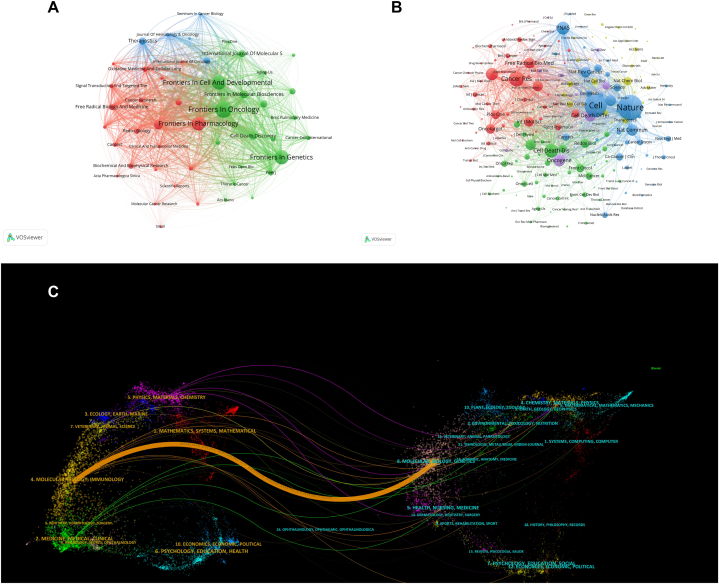
Visualization of contributing journals (A), co-cited journals (B), and the dual-map overlay of journals (C) in ferroptosis research within lung cancer.

The analysis of ferroptosis literature in lung cancer revealed a concentration of research output in a select number of scientific journals. Table 2 lists the top 10 productive journals in this field, with *Frontiers In Oncology* leading with 19 publications, accounting for 3.89 % of the total literature, followed by *Frontiers In Cell And Developmental Biology* and *Frontiers In Pharmacology*, each with 16 publications (Fig. 4A). The impact factors (IFs) of these journals, as reported in JCR2022, range from 3.7 to 12.4, with Theranostics having the highest IF. The majority of these journals are ranked within the first and second quartiles of the JCR, indicating their influential status.

**Table 2:** The top 10 productive journals related to ferroptosis in lung cancer.

Rank	Journal	Publications(%)	IF(JCR2022)	JCR Quartile
1	Frontiers In Oncology	19(3.89 %)	4.7	Q2
2	Frontiers In Cell And Developmental Biology	16(3.28 %)	5.5	Q1
3	Frontiers In Pharmacology	16(3.28 %)	5.6	Q1
4	Frontiers In Genetics	16(3.28 %)	3.7	Q2
5	Cell Death & Disease	10(2.05 %)	9	Q1
6	Frontiers In Molecular Biosciences	9(1.84 %)	5	Q2
7	Cell Death Discovery	8(1.64 %)	7	Q2
8	Theranostics	8(1.64 %)	12.4	Q1
9	International Journal of Molecular Sciences	8(1.64 %)	5.6	Q1
10	Cells	8(1.64 %)	6	Q2

Co-citation analysis reveals *Nature* as the most cited journal (1273 citations), followed by *Cell* (1106 citations), underscoring their pivotal roles in disseminating ferroptosis research (Fig. 4B–Table 3). The top-cited journals' IFs are notably high, with *Nature* at an IF of 64.8 and *Cell* at 64.5, both in the first quartile of JCR. The correlation between high IFs and citation frequency underscores the importance of these journals in advancing ferroptosis research in lung cancer.

**Table 3:** Top 10 journals for co-citation of ferroptosis in lung cancer.

Rank	Cited journal	Citation	IF(JCR2022)	JCR Quartile
1	Nature	1273	64.8	Q1
2	Cell	1106	64.5	Q1
3	Cancer Research	675	11.2	Q1
4	Cell Death Discovery	578	7	Q2
5	PNAS	530	11.1	Q1
6	Journal of Biological Chemistry	508	4.8	Q2
7	Oncogene	487	8	Q1
8	Cell Death and Differentiation	461	12.4	Q1
9	Natur Communications	453	16.6	Q1
10	Free Radical Biology and Medicine	447	7.4	Q1

The dual-map overlay of journals in Fig. 4C illustrates the relationship between citing and cited journals, with articles in the Molecular/Biology/Genetics domain primarily cited by those in Molecular/Biology/Immunology. This mapping effectively represents the interconnectedness of these research fields.

### 3.4 Authors and Co-cited authors

**Fig. 5:**
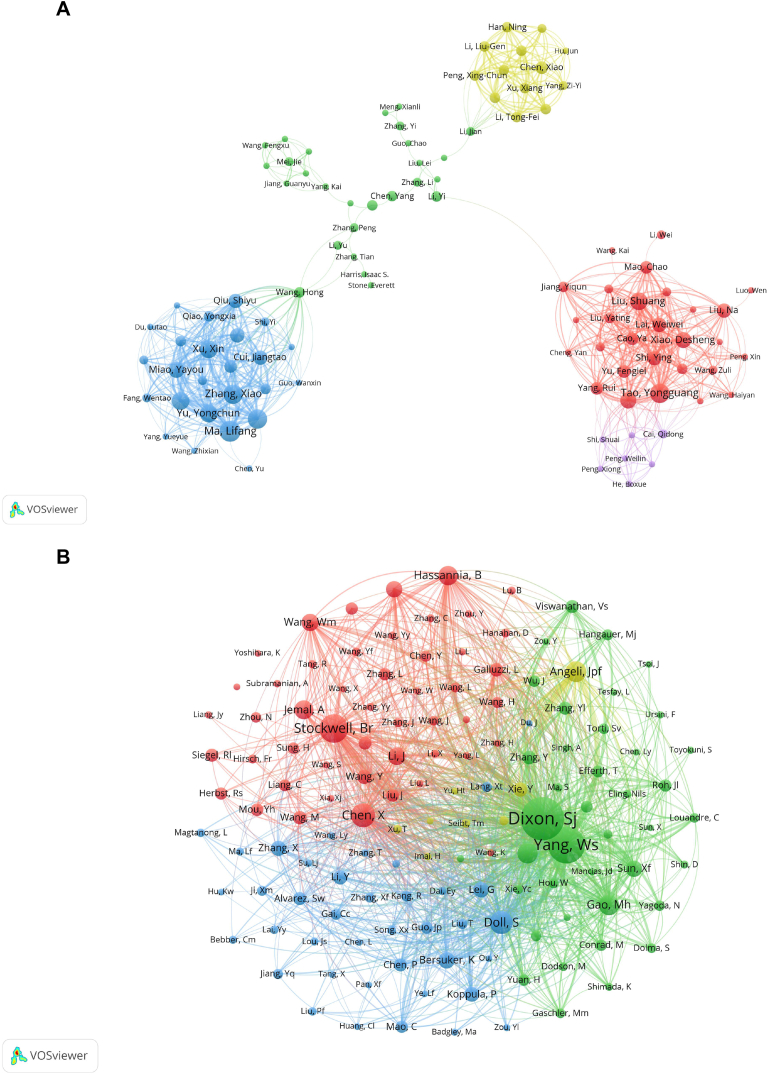
Visualization of authors (A) and co-cited Authors (B) on research of ferroptosis in lung cancer.

The bibliometric analysis mapped the landscape of ferroptosis research in lung cancer, identifying the most prolific authors and the most frequently co-cited authors (Fig. 5A and B). Table 4 presents the top 10 authors by publication count, with Lifang Ma at the forefront with 10 publications. Notably, all these leading authors are based in China, reflecting the country's significant contributions to the field.

**Table 4:** Top 10 authors and co-cited authors on research of ferroptosis in lung cancer.

Rank	Authors	Location	Count	Co-Cited Authors	Location	Citations
1	Lifang Ma	China	10	Scott J Dixon	USA	383
2	Yongguang Tao	China	9	Wan Seok Yang	USA	295
3	Xiao Zhang	China	9	Brent R Stockwell	USA	197
4	Jiayi Wang	China	9	Xin Chen	China	148
5	Shuang Liu	China	8	Sebastian Doll	Germany	143
6	Xiaoting Tian	China	8	Jose Pedro Friedmann Angeli	Germany	117
7	Yongchun Yu	China	8	Minghui Gao	USA	116
8	Xin Xu	China	8	Le Jiang	USA	114
9	Xiang Wang	China	7	Behrouz Hassannia	Belgium	108
10	Yayou Miao	China	7	Ahmedin Jemal	USA	104

The study also revealed the most co-cited authors, with Scott J Dixon leading the list with 383 citations. This indicates the significant influence of Dixon's work in the field of ferroptosis in lung cancer research. The co-cited authors mainly hail from the USA, with others from Germany, China, and Belgium, highlighting the global nature and collaborative spirit of the research.

### 3.5 Co-cited references and reference with citation bursts

Our analysis successfully identified the most influential studies in ferroptosis and lung cancer research, as evidenced by co-citation frequency. Table 5 lists the top 10 co-cited references, with Scott J Dixon's 2012 *Cell* article, “Ferroptosis: an iron-dependent form of nonapoptotic cell death,” leading with 447 citations. This seminal work has significantly influenced subsequent research in this field.

**Table 5:** Ranking of the top 10 co-cited references for ferroptosis in lung cancer.

Rank	Reference	Citation	Year	First Author	Journal
1	Ferroptosis: an iron-dependent form of nonapoptotic cell death	447	2012	Scott J Dixon	Cell
2	Regulation of ferroptotic cancer cell death by GPX4	398	2014	Wan Seok Yang	Cell
3	Ferroptosis: A Regulated Cell Death Nexus Linking Metabolism, Redox Biology, and Disease	299	2017	Brent R Stockwell	Cell
4	Ferroptosis as a p53-mediated activity during tumor suppression	262	2015	Le Jiang	Nature
5	CD8^+^ T cells regulate tumor ferroptosis during cancer immunotherapy	258	2019	Weimin Wang	Nature
6	Targeting Ferroptosis to Iron Out Cancer	258	2019	Behrouz Hassannia	Cancer Cell
7	The CoQ oxidoreductase FSP1 acts parallel to GPX4 to inhibit ferroptosis	256	2019	Kirill Bersuker	Nature
8	FSP1 is a glutathione-independent ferroptosis suppressor	237	2019	Sebastian Doll	Nature
9	Broadening horizons: the role of ferroptosis in cancer	236	2021	Xin Chen	Nature Reviews Clinical Oncology
10	Activation of the p62-Keap1-NRF2 pathway protects against ferroptosis in hepatocellular carcinoma cells	234	2016	Xiaofang Sun	Hepatology

**Fig. 6:**
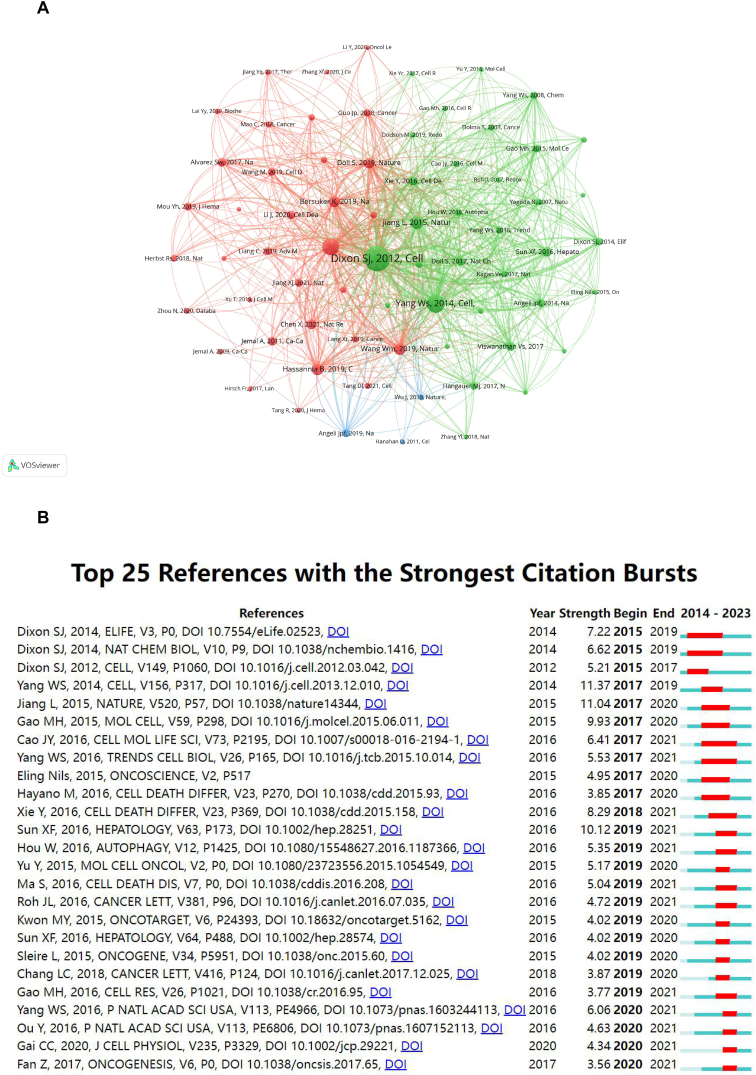
Visualization of co-cited references (A) and the top 25 references with strong citation bursts (B) on research of ferroptosis in lung cancer.

Predominantly, these leading articles were published in prestigious journals such as *Cell*, *Nature*, and *Nature Reviews Clinical Oncology*, with *Cell* notably including three articles in the top ten (Fig. 6A). These publications encompass a range of topics related to ferroptosis, including its regulation, molecular mechanisms, and implications in tumor suppression and cancer therapy.

Most of these key articles were published in prestigious journals like *Cell*, *Nature*, and *Nature Reviews Clinical Oncology*, indicating the importance of these platforms in the dissemination of ferroptosis research. The range of topics these articles cover includes ferroptosis regulation, molecular mechanisms, and its role in tumor suppression and cancer therapy.

The “citation burst” analysis identified articles with a sudden increase in citations over a period, signifying emerging high-profile research topics [23]. Our analysis identified the top 25 references with the most significant citation bursts (Fig. 6B), highlighting a rapidly growing interest in this field from 2014 to 2023. The paper “Regulation of ferroptotic cancer cell death by GPX4” by Wan Seok Yang exhibited the strongest citation burst (11.37) among these references, with bursts occurring from 2017 to 2019 [24]. Additionally, three articles by Dixon S.J. stood out for their impactful citation frequency and sustained influence from 2015 to 2019 [3,25,26].

### 3.6 Hotspots and frontiers

Utilizing VOSviewer and CiteSpace, we analyzed 986 author keywords from 488 documents, with 71 meeting the minimum document criterion (at least four documents per keyword).

**Fig. 7:**
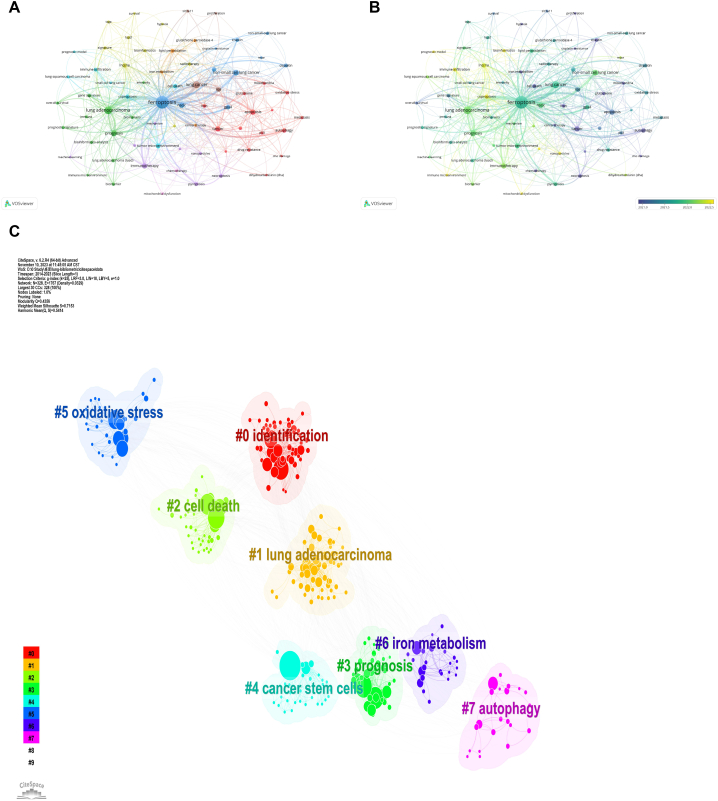
Visualization of keywords (A), temporal map of keywords (B), and keyword clusters (C) on research of ferroptosis in lung cancer.

The bibliometric analysis, incorporating keyword co-occurrence and temporal progression maps, provided insightful perspectives on the evolving dynamics of ferroptosis research in lung cancer. The term “ferroptosis” emerged as a central node in the co-occurrence network, indicating its frequent mention in the literature. Surrounding this central node are significant terms like “apoptosis,” “immunotherapy,” “tumor microenvironment,” “ROS,” and “GPX4,” highlighting these as foundational and recurrent topics in the field (Fig. 7A).

The progression map provides temporal insights, highlighting emerging research interests (Fig. 7B). Keywords such as “cuproptosis”, “prognostic model”, “bioinformatics”, “immune infiltration”, “nanoparticles”, “immune microenvironment”, and “mitochondrial dysfunction” are marked in a yellowish hue, indicating their status as cutting-edge and evolving research areas. In contrast, terms displayed in a purple hue, such as “apoptosis” and “oxidative stress,” are recognized as foundational components of the existing research corpus, providing a historical backdrop for current trends.

Fig. 7C identifies eight relevant clusters, ranging from #0 (identification) to #7 (autophagy), as delineated by CiteSpace. The modularity (Q) value and silhouette (S) value are employed in CiteSpace to evaluate the robustness of clustering. Generally, a Q value above 0.3 and an S value greater than 0.7 indicate reliable and noteworthy clustering. Our cluster analysis resulted in a Q value of 0.44 and an S value above 0.7, reinforcing the validity and significance of these clusters.

## 4 Discussion

This study utilized VOSviewer (version 1.6.19), CiteSpace (version 6.2.R4), and the R package “bibliometrix” (version 3.2.1) for a detailed analysis of 488 articles on ferroptosis in lung cancer from 2014 to October 1, 2023. Our analysis of data from the WOSCC identified key spatial and temporal trends, influential authors, seminal articles, emergent hotspots, and the frontiers of this field.

## 5 General information

The increasing number of annual publications, depicted in Fig. 2, indicates a rising academic interest in ferroptosis in lung cancer. The period from 2014 to 2018 showed modest publication volumes, marking the field's nascent stage. In contrast, the period from 2019 to 2023 witnessed a significant surge in research activity, signifying major advancements and enhanced academic engagement.

### 5.1 Geographical and institutional distribution

Geographical analysis (refer to Fig. 3, Fig. 4) reveals China's dominant role in ferroptosis research concerning lung cancer. China's high centrality index underscores its influential and integrated position within the global research network, likely driven by targeted research priorities and funding. However, the comparatively lower centrality indices of individual Chinese institutions suggest a widespread distribution of research efforts across multiple organizations. The United States and Germany's contributions emphasize the field's global appeal and collaborative potential. Contributions from other countries, including South Korea, India, Spain, Belgium, and Australia, reflect a diverse but less central role in the research landscape. Notably, Australia's null centrality index, despite its comparable publication numbers to certain European countries, suggests a less integrated role in the global context. This diverse international participation enriches the research output, fostering a comprehensive understanding of ferroptosis in lung cancer from various perspectives and methodologies.

### 5.2 Status and quality of journals, authors, and references

The predominance of high-impact journals among the most cited and productive in ferroptosis research highlights the field's importance (see Table 2, Table 3). The correlation between journal impact factors and citation frequencies underscores the seminal works' role in advancing scientific discovery and discourse. High-impact journals play a critical role in disseminating key findings, crucial for furthering our understanding of ferroptosis and its implications for lung cancer therapy.

The concentration of publications in select journals suggests the emergence of specialized knowledge clusters within the scientific community, promoting focused discussions and rapid advancements in understanding ferroptosis. However, this also raises concerns about the diversity of publication practices and the accessibility of high-quality research across different platforms.

Research on ferroptosis in lung cancer is predominantly published in Frontiers series journals, indicating their popularity in this research domain. *Theranostics* (IF = 12.4, Q1) and *Cell Death & Disease* (IF = 9, Q1) are among the journals with the highest impact factors. Regarding the co-cited journals, we could find most of them are high-impact Q1 journals. Obviously, these journals are high-quality international journals, providing support for the study of ferroptosis in lung cancer. What's more, the current focus on molecular, biology, and immunology journals, with fewer publications in clinical journals, suggests that much of the research remains at the basic research stage.

As the field evolves, it is imperative to monitor how publication patterns impact the research landscape of ferroptosis in lung cancer. The introduction of new journals, changes in impact factors, and shifts in research funding focus could significantly influence future publication and citation trends. Researchers are encouraged to contribute to high-impact journals while also engaging with a broader range of publication outlets to maintain diverse and comprehensive scientific discourse.

In conclusion, this network visualization acts as both a map of current scientific endeavors and a guide for future research directions. Understanding collaboration patterns and information flow is crucial for researchers navigating the complex landscape of ferroptosis in lung cancer. Deepening our understanding and fostering interdisciplinary connections are essential to unlocking the potential of ferroptosis-targeted therapies.

Regarding co-cited authors, Scott J. Dixon, with 383 citations, emerges as the most frequently cited author, followed by Wan Seok Yang (295 citations) and Brent R. Stockwell (197 citations). Dixon's seminal 2012 publication, “Ferroptosis: an iron-dependent form of nonapoptotic cell death” in Cell, has been pivotal in defining and characterizing ferroptosis. The paper explores the distinct biochemical and genetic markers of ferroptosis, distinguishing it from traditional apoptosis and necrosis, and examines its reliance on iron and the role of reactive oxygen species (ROS). It also discusses the broader implications of ferroptosis in various diseases and its potential in novel therapeutic approaches [3]. In subsequent studies, Dixon elaborated on the interplay between iron and ROS in cell death, delving into the ramifications of iron overload and ROS in neurodegenerative diseases, cancer, and other pathologies [25]. Additionally, Dixon's 2014 article in Elife summarizes research on inhibiting the cystine-glutamate exchange system x_c_− and inducing ferroptosis through agents like erastin and sorafenib, investigating genetic resistance factors and clinical implications [26]. These contributions by Dixon have laid a solid theoretical and experimental foundation for ferroptosis research in lung cancer, with his high citation count reflecting significant impact and recognition within the scientific community. His leading position in citation frequency suggests that his crucial role in shaping the current trajectory of lung cancer ferroptosis research.

Significant citation counts for authors like Brent R. Stockwell and Wan Seok Yang underscore the importance of their contributions to the understanding of ferroptosis mechanisms and clinical applications in lung cancer treatment. Their work, along with that of other notable researchers, provides valuable insights into the evolution and dynamics of this field.

Monitoring shifts in authorship is vital as emerging researchers contribute to the field and established authors expand their influence. Understanding the dynamics of publication and citation networks provides insights into the evolution of ferroptosis research in lung cancer, identifying areas for further investigation and collaboration.

This bibliometric analysis can guide future collaborative efforts, indicating which researchers are central to the field and might be valuable collaborators. Additionally, identifying influential authors and their works assists new researchers in quickly familiarizing themselves with the core literature and ongoing dialogues within the field.

A co-cited reference is a reference that is cited together by multiple other publications, serving as a foundational element within a field [27]. In this study, the top 10 co-cited references were identified to determine the research basis of ferroptosis in lung cancer. Scott J. Dixon et al.'s most co-cited 2012 study, previously discussed, will not be reiterated here. Wan Seok Yang's 2014 paper in *Cell*, “Regulation of ferroptotic cancer cell death by GPX4,” discusses ferroptosis's relevance in cancer research. It explores the role of the enzyme GPX4 in preventing lipid peroxidation and highlights the potential therapeutic applications of ferroptosis-inducing compounds in cancer treatment. The study delves into the mechanisms and factors involved in ferroptosis, shedding light on its significance in cancer cell sensitivity and tumor growth inhibition [24]. Brent R. Stockwell, also the corresponding author of the previously mentioned article, published the third most co-cited paper in *Cell* in 2017 [28]. This review provides a comprehensive overview of ferroptosis, covering its mechanisms, cellular pathways, and disease relevance. It reviews the role of genes, proteins, and metabolic pathways in regulating ferroptosis, highlighting its therapeutic potential in various conditions.

Overall, the top 10 co-cited references emphasize the convergence of metabolic, redox, and disease-related pathways in ferroptosis, underlining its critical role in cellular fate determination. These studies collectively form a robust evidence base, positioning ferroptosis as a fundamental process with significant implications for biology and medicine. Advancing our knowledge of ferroptosis promises to revolutionize disease treatment strategies and enhance our understanding of cellular life and death.

### 5.3 Hotspots and frontiers

The prominent feature of “ferroptosis” within the network underscores its significant impact on lung cancer research. A recent shift toward terms such as “cuproptosis” and “mitochondrial dysfunction” indicates an expansion in the cell death research paradigm, exploring novel mechanisms and their implications in cancer pathophysiology.

The yellow hue associated with terms like “prognostic model,” “bioinformatics,” “immune infiltration,” and “nanoparticles” highlights an interdisciplinary approach to research. This suggests that the integration of computational analytics, immune profiling, and advanced materials science is propelling the understanding and application of ferroptosis in lung cancer.

The emphasis on the “immune microenvironment” in recent research underscores its growing recognition as a crucial factor in cancer progression and response to therapy. It suggests that modulating the immune environment could be a key strategy for utilizing ferroptosis therapeutically.

In contrast, the foundational research areas such as “apoptosis” and “oxidative stress,” marked by a purple hue, reflect their historical significance and foundational role in shaping the current research directions. This base provides essential insights into cell death mechanisms that have informed the current focus on ferroptosis and its nuances.

In summary, the bibliometric analysis unveils a dynamic and evolving research landscape where past research on apoptosis and oxidative stress has set the stage for current explorations into ferroptosis, its mechanisms, and implications in lung cancer. The integration of novel computational methods, understanding of the immune microenvironment, and advancements in nanotechnology are leading to a deeper comprehension of ferroptosis and opening new therapeutic avenues in the fight against lung cancer.

The study has categorized keywords into eight principal clusters, as illustrated in Fig. 7C. To facilitate a comprehensive summary and synthesis, these clusters have been integrated into broader thematic categories:

1 Lung adenocarcinoma specifics (cluster #1 lung adenocarcinoma):

The distinct cluster dedicated to lung adenocarcinoma reflects a targeted focus within the research community on this particular cancer subtype. Lung adenocarcinoma is characterized by a variety of driver mutations and a unique pathological profile that differentiates it from other forms of lung cancer [29]. Studies within this cluster address the role of ferroptosis in the context of adenocarcinoma-specific pathways, such as those involving EGFR mutations, ALK rearrangements, and KRAS mutations. The cluster's isolation in the analysis underscores the importance of considering the specificities of adenocarcinoma in developing ferroptosis-based therapies. Discussions would also delve into how ferroptosis induction or inhibition could influence the tumor microenvironment, affect drug delivery and uptake, and modulate the immune response, particularly tailored to the biological makeup of lung adenocarcinoma. As personalized medicine advances, understanding ferroptosis within this context could lead to highly individualized and potentially more effective treatment regimens for patients with this subtype.

2 Biomarker Identification and Prognosis (clusters #0 identification, #3 prognosis):

The convergence of the identification and prognosis clusters signifies a crucial trend in lung cancer research: the search for reliable biomarkers. Identifying ferroptosis markers could significantly enhance early detection, vital since lung cancer often presents asymptomatically until advanced stages [30]. These biomarkers' predictive value could also inform treatment choices and anticipate therapeutic responses. This dual focus on identification and prognosis necessitates a multidisciplinary approach, combining molecular biology, bioinformatics, and clinical sciences, to translate these biomarkers from research to clinical practice. For patients, this could mean transitioning towards more proactive and precise care, with therapies tailored to the ferroptotic profile of their tumors.

3 Cellular Death Mechanisms and Metabolic Regulation (clusters #2 cell death, #5 oxidative stress, #6 iron metabolism, #7 autophagy):

The amalgamation of clusters related to cell death and metabolism emphasizes the complexity of ferroptosis and its regulatory mechanisms. Ferroptosis, an iron-dependent form of non-apoptotic cell death, is characterized by lethal lipid peroxide accumulation, closely tied to cellular metabolism and redox biology [28]. The interaction between oxidative stress and iron metabolism is particularly significant, as disturbances in iron homeostasis can lead to increased reactive oxygen species production, propelling the cell towards death [31]. Autophagy's role in this context is dual: it can aid cell survival by degrading damaged organelles and proteins or facilitate cell death by degrading essential survival components [32]. Discussions in this theme would critically examine how lung cancer cells bypass these death mechanisms and how therapies could be designed to restore or exploit these pathways. Additionally, ferroptosis's metabolic aspects open discussions on cancer cells' vulnerability to metabolic stress, potential metabolic targeting in therapy, and implications for drug resistance.

4 Cancer Stem Cells and Therapeutic Resistance (cluster #4 cancer stem cells):

The cluster focusing on cancer stem cells (CSCs) addresses a pivotal challenge in cancer treatment: the eradication of the cell population responsible for recurrence and metastasis. CSCs are known for their resistance to conventional therapies, partly due to their quiescent nature and efficient DNA repair mechanisms [33]. The discussion here would explore the potential of ferroptosis to selectively target CSCs, given that these cells might exhibit distinct susceptibilities to iron dysregulation and lipid peroxidation. Leveraging ferroptosis against CSCs could represent a novel therapeutic strategy that not only complements existing treatments but also addresses the problem of minimal residual disease [34]. The challenge lies in identifying specific triggers or vulnerabilities of CSCs that can be modulated to induce ferroptosis without harming normal stem cells. This discussion would extend into the broader implications for therapy, considering how ferroptosis could be integrated into treatment protocols to overcome resistance and prevent relapse, thereby improving the long-term outcomes for patients with lung cancer.

These themes, derived from the cluster analysis, provide unique insights into the role of ferroptosis in lung cancer, presenting diverse challenges and opportunities. Collectively, they highlight the need for a multifaceted approach in lung cancer research and treatment. By exploring the role of ferroptosis in various aspects of lung cancer biology, novel strategies can be developed to combat this disease. The potential of ferroptosis as a therapeutic target in lung cancer is promising and warrants further exploration and validation in clinical settings.

Building on the comprehensive analysis in previous sections, this paper proposes several promising directions for future development in the field of ferroptosis in lung cancer. These directions aim to deepen our understanding and foster innovative therapeutic approaches:

1. **Elucidating the Epigenetic Regulation of Ferroptosis in Lung Cancer:** This proposal advocates for a groundbreaking study into the epigenetic mechanisms governing ferroptosis, examining the roles of DNA methylation, histone modification, and non-coding RNAs in predisposing lung cancer cells to ferroptotic death. This research could reveal novel epigenetic targets, offering potential for priming cancer cells towards ferroptosis.
2. **Single-Cell Sequencing to Dissect Ferroptosis Heterogeneity:** The study recommends employing single-cell RNA sequencing to dissect the variability in ferroptosis response among tumor cell populations, aiming to identify subpopulations with distinct sensitivities to ferroptosis. This could lead to targeted therapy approaches based on single-cell analyses. This approach could identify cancer cell subpopulations particularly susceptible to ferroptosis, enabling the development of more precise and effective therapeutic strategies.
3. **AI-Driven Drug Discovery for Ferroptosis Inducers:** The study recommends employing single-cell RNA sequencing to dissect the variability in ferroptosis response among tumor cell populations, aiming to identify subpopulations with distinct sensitivities to ferroptosis. This could lead to targeted therapy approaches based on single-cell analyses. This approach could identify cancer cell subpopulations particularly susceptible to ferroptosis, enabling the development of more precise and effective therapeutic strategies.
4 **Crispr-Cas9 screens to identify ferroptosis modulators:** implement implementing CRISPR-Cas9 genetic screens is suggested to discover novel genes that modulate ferroptosis in lung cancer cells. This high-throughput technique could uncover unexpected genetic determinants of ferroptosis and potential therapeutic targets to trigger cell death in lung cancer.
5. **Multi-omic Integration to Profile Ferroptosis Pathways:** A multi-omic integration strategy combining genomics, proteomics, metabolomics, and lipidomics is advocated to construct comprehensive profiles of ferroptosis pathways in lung cancer. This approach would provide a deeper understanding of the ferroptotic process and its regulatory networks.
6. **Tumor Microenvironment Engineering to Induce Ferroptosis:** Novel approaches are suggested to engineer the tumor microenvironment to selectively induce ferroptosis in lung cancer cells. This could involve modulating the immune milieu or stromal interactions to enhance ferroptotic signaling, potentially synergizing with immunotherapies.
7. **Investigating Ferroptosis in Circulating Tumor Cells (CTCs):** Studying ferroptosis in CTCs is proposed as a real-time liquid biopsy marker for lung cancer progression and treatment response. This research direction could lead to non-invasive prognostic and diagnostic tools reflecting dynamic changes in tumor biology.
8. **Ferroptosis and Metabolic Flexibility in Lung Cancer:** Exploring metabolic flexibility and its impact on ferroptosis in lung cancer, with a focus on how metabolic reprogramming can sensitize or confer resistance to cancer cells against ferroptosis, offers a novel perspective to target metabolic vulnerabilities.

In summary, these proposed directions build on the rich insights gained from the bibliometric analysis, aiming to deepen our understanding and inspire new therapeutic interventions targeting ferroptosis in lung cancer. The potential for ferroptosis as a therapeutic avenue is immense, offering promising prospects for advancing lung cancer treatment.

### 10.1 Limitations

While this study provides a comprehensive visual bibliometric analysis of ferroptosis in lung cancer research up to October 1, 2023, it is not without its limitations. Bibliometric analysis, by nature, focuses on recent studies and thus offers a temporal snapshot that might change as the field evolves. Therefore, regular updates are essential to capture the latest trends in ferroptosis and lung cancer research. Another limitation is the exclusive use of the WOSCC database, which may overlook relevant studies from other databases such as PubMed, Cochrane Library, and Google Scholar. The focus on English-language publications introduces a potential bias, potentially excluding significant non-English scholarly contributions. Additionally, dataset inconsistencies, like variable institutional naming, might impact the precision of our analysis. It is also crucial to acknowledge the capabilities and limitations of the employed bibliometric tools—VOSviewer, CiteSpace, and the R package “bibliometrix"—as data gaps may exist despite their use.

## 11 Conclusion

Our extensive bibliometric analysis, covering nearly a decade of ferroptosis in lung cancer research, has illuminated significant insights and emerging trends that signify a paradigm shift in understanding and treating this challenging disease. The substantial increase in research output from 2019 to 2023 highlights the escalating recognition of ferroptosis as a critical area in cancer biology. The involvement of high-impact publications and influential authors like Scott J. Dixon emphasizes the quality and impact of research in this field.

The identification of key thematic areas – Lung Cancer Specifics; Biomarker Identification and Prognosis; Cellular Death Mechanisms and Metabolic Regulation; and Cancer Stem Cells and Therapeutic Resistance – offers a structured foundation for ongoing and future research. The trend towards investigating aspects like the immune microenvironment and mitochondrial dysfunction suggests an expanding scope in ferroptosis research.

Our analysis also underscores the global collaborative nature of this field, showcasing significant contributions from various regions. This collaboration is essential for developing a comprehensive understanding of ferroptosis in lung cancer and translating research findings into clinical applications.

Looking ahead, the potential of ferroptosis as a therapeutic target in lung cancer is clear. Integrating ferroptosis-based strategies with current treatments could lead to more effective, personalized therapies. By continuing to delve into the multifaceted nature of ferroptosis in lung cancer, we are laying the groundwork for innovative treatments that could markedly improve patient outcomes. This study not only consolidates existing knowledge but also spurs ongoing research, opening new avenues in the fight against lung cancer.

### Data availability statement

Data associated with our study has not been deposited into any publicly available repository. The original contributions presented in the study are included in the article, and further inquiries can be directed to the corresponding authors.

## Funding

This work was financially supported by the Scientific Research Launch Project for new employees of the Second Xiangya Hospital of Central South University (to ZZ)

## CRediT authorship contribution statement

**Huatao Zhou:** Writing – original draft, Software, Formal analysis, Data curation, Conceptualization. **Yu Mao:** Writing – review & editing, Software. **Zijing Zhou:** Writing – review & editing, Supervision, Investigation, Funding acquisition, Conceptualization.

## Declaration of competing interest

The authors declare that they have no known competing financial interests or personal relationships that could have appeared to influence the work reported in this paper.

## Nonstandard Abbreviations and Acronyms

WOSCC: Web of Science Core Collection
JCR: Journal Citation Reports
IFs: Impact Factors
ROS: Reactive Oxygen Species
CSCs: Cancer Stem Cells
CTCs: Circulating Tumor Cells
